# Vitamin D in children with growth hormone deficiency due to pituitary stalk interruption syndrome

**DOI:** 10.1186/s12887-018-0992-3

**Published:** 2018-01-24

**Authors:** Cécile Delecroix, Raja Brauner, Jean-Claude Souberbielle

**Affiliations:** 1 Fondation Ophtalmologique Adolphe de Rothschild and Université Paris Descartes, Paris, France; 2 Assistance Publique-Hôpitaux de Paris, Hôpital Necker-Enfants Malades, Service d’Explorations Fonctionnelles, Paris, France

**Keywords:** 1,25(OH)_2_D, 1,25-dihydroxyvitamin D, 25OHD, 25-hydroxyvitamin D, Growth hormone, Growth hormone deficiency, Insulin-like growth factor 1, Pituitary stalk interruption syndrome, Vitamin D

## Abstract

**Background:**

Recent studies have shown a relationship between vitamin D status and growth hormone (GH) and insulin-like growth factor 1 (IGF1). The objective of this study was to assess vitamin D status in children with GH deficiency due to pituitary stalk interruption syndrome (PSIS) and to investigate the relationship between 25-hydroxyvitamin D (25OHD) and 1,25-dihydroxyvitamin D (1,25 (OH) _2_D) serum levels and patient characteristics.

**Methods:**

A retrospective single-center study of 25OHD and 1,25(OH)_2_D serum concentrations in 50 children with PSIS at the initial evaluation before treatment.

**Results:**

Mean concentrations of 33.2 ± 18.0 ng/mL for 25OHD and 74.5 ± 40.7 ng/L for 1,25(OH)_2_D were measured. Additionally, 25OHD concentrations were significantly higher in boys than in girls (*p* = 0.04) and lower in the cold season than in the sunny season (*p* = 0.03). Significant positive correlations were observed between the GH peak and serum 1,25 (OH) _2_D concentrations (Rho = 0.35; *p* = 0.015) and the 1,25(OH)_2_D/25OHD ratio (Rho = 0.29; *p* < 0.05). No correlation was found for other characteristics, including IGF1.

**Conclusions:**

Vitamin D status in children with hypothalamic-pituitary deficiency due to PSIS was similar to that reported in national and European studies in healthy children. The positive significant correlations between the GH peak and the 1,25 (OH)_2_D concentration as well as with the 1,25 (OH)_2_D/25OHD ratio suggest that even in these patients who had severely impaired GH secretion and low IGF1 levels, an interplay between the GH/IGF1 axis and the vitamin D system still exists.

## Background

Growth hormone (GH) deficiency (GHD) can be congenital or acquired. Pituitary stalk interruption syndrome (PSIS), a sign of congenital and permanent GHD, is an anomaly of the pituitary gland characterized by a combination of specific findings on magnetic resonance imaging, including an interrupted pituitary stalk, an absent or ectopic posterior pituitary and anterior pituitary hypoplasia [1]. Because of the heterogeneity in clinical, biological and imaging presentations, recent efforts have focused on discriminating patients with multiple hypothalamic-pituitary (HP) deficiencies from those with isolated GHD [2]. Previous studies have shown an association between PSIS and other syndromes and/or malformations, and occurrence of familial forms have led to identification of gene mutations [3–7].

Humans mainly acquire vitamin D from exposure to sunlight, but it can also be obtained from food (oily fish). Vitamin D is first metabolized to 25-hydroxyvitamin D (25OHD) in the liver and then to its active form, 1,25-dihydroxyvitamin D (1,25(OH)_2_D), in the kidneys. Overall, the concentration of serum 25OHD reflects the vitamin D status of an individual. Although debate remains regarding the 25OHD level that defines the optimal level, vitamin D deficiency is considered to be common worldwide in both adults and children [8–11]. Vitamin D has a well-known role in bone development in utero and in childhood, and it maintains bone health in adults through effective calcium regulation and bone mineralization [12]. Because vitamin D receptors are present in many tissues, vitamin D may also have “non-classical” effects. Indeed, vitamin D deficiency has been associated with obesity in children [13], cardiovascular disease, type 2 diabetes, cancers (colon, prostate or breast) and auto-immune disorders [14–16].

Recent studies have shown a relationship between vitamin D status and GH and insulin-like growth factor 1 (IGF1). For example, initiation of cholecalciferol treatment increases serum IGF1 levels in children and adults with vitamin D deficiency [17, 18], and Wei et al. and Saggese et al. showed that six and 12 months of GH treatment, respectively, raised 1,25(OH)_2_D concentrations in children with GHD [19, 20].

The first objective of the present study was to assess vitamin D status at the initial evaluation before GH treatment in a large series of children with GHD due to PSIS attended by a single endocrinologist. The second objective was to investigate the relationship between 25OHD and 1,25 (OH) _2_D serum levels and patient characteristics, mainly the GH peak and serum IGF1 level.

## Methods

### Patients

**Fig. 1:**
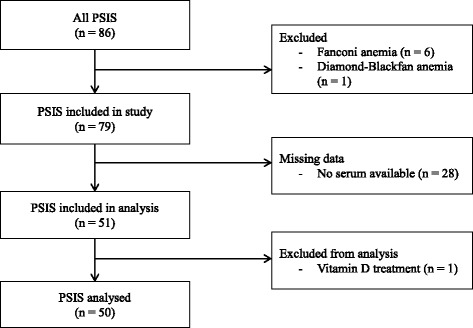
Flowchart of the inclusion in the study

This retrospective single-center study included 50 children (28 boys and 22 girls) of a total of 86 (58%) PSIS patients monitored for HP deficiency by a senior pediatric endocrinologist (R. Brauner) between 1982 and 2016 and for whom a serum sample preserved at the initial evaluation at − 22 °C was still available. Patients with a syndrome that can interfere with their growth rate, i.e., Fanconi or Diamond-Blackfan anemia, were excluded (Fig. 1). At the initial evaluation, the characteristics of the 50 patients were similar to those of the 36 without available samples (Table 1). The criterion for GHD was a GH peak response of less than 20 mU/L or 6.7 ng/mL after two pharmacological stimulation tests.

**Table 1:** Characteristics of the included and excluded patients with PSIS

	All PSIS		PSIS included		PSIS excluded		*p*
	(*n* = 86)		(*n* = 50)		(*n* = 36)		
Boys	54	63%	28	56%	26	72%	0.9
Girls	32	37%	22	44%	10	28%	
Isolated GH deficiency	39	47%	22	44%	17	47%	0.8
Multiple HP deficiency	47	43%	28	56%	19	53%	
	Mean ± SD		Mean ± SD		Mean ± SD		
Age at diagnosis, years	4.9 ± 4.8		5.4 ± 4.9		4.2 ± 4.2		0.1
Bone age, years	3.7 ± 3.9		3.3 ± 4.1		3.8 ± 3.5		0.8
Height, SDS	−3.1 ± 1.5		−2.8 ± 1.1		−3.2 ± 1.8		0.7
BMI, SDS	0.2 ± 2.8		0.2 ± 2.2		0.2 ± 3.4		0.2
Growth rate, SDS	− 2.7 ± 1.4		−2.8 ± 1.4		−3 ± 1.4		0.6
GH peak, mU/L	5.9 ± 6.7		6.3 ± 6.0		4.1 ± 4.6		0.3
IGF1, SDS	− 3.6 ± 1.5		−3.6 ± 1.6		−3.6 ± 1.1		0.8
Free thyroxin, pmol/L	12.3 ± 7.8		12.1 ± 7.8		12.6 ± 10.8		0.5

### Methods

The following data were collected at the initial clinical evaluation: age, height, growth rate during the previous year and body mass index (BMI), all expressed in standard deviation scores (SDS) according to chronological age [21, 22]. Bone age was assessed by R. Brauner according to the Greulich and Pyle method [23]. The initial biological evaluation included the IGF1 plasma concentration (expressed in ng/mL and SDS according to age and pubertal stage) and the GH peak level after a stimulation test (ornithine, arginine-insulin and/or glucagon test) or during spontaneous hypoglycemia in a few neonates. As we used various GH assays over the study period, we expressed the GH peak concentration in mU/L using conversion factors (ng/mL ⇔ mU/L) that were specific of the international standard used to calibrate the GH assay (i.e., 1 ng/mL = 2 mU/L for the assay calibrated with the old 66/217 international standard composed of poorly purified pituitary GH; 1 ng/mL = 2.6 mU/L for the assay calibrated against the 80/505 international standard composed of highly purified pituitary GH; 1 ng/mL = 3 mU/L for the assay calibrated against the 98/574 international standard composed of recombinant human (h)GH).

Multiple HP deficiency was diagnosed by GH deficiency in association with one or multiple deficiencies in the following: thyroid-stimulating hormone, adrenocorticotropic hormone, and/or luteinizing hormone and follicle-stimulating hormone [2]. Pituitary functions other than GH were evaluated by basal blood cortisol at 08.00 h, free thyroxin, prolactin, and the thyroid stimulating hormone response to thyrotropin releasing hormone; the plasma and urinary osmolalities after water deprivation for 12-h were normal in the first 29 patients evaluated, showing a normal concentration capacity. The normal limits were 12 to 28 pmol/L for plasma free thyroxin, and 0.6 to 4 mU/L for basal plasma thyroid stimulating hormone, 14 ± 7 mU/L for its peak, and < 10 mU/L for its value at 120 min after the thyrotropin releasing hormone test; and 5–25 μg/L for basal prolactin, except for neonates, who had higher concentrations. Adrenocorticotropic deficiency was diagnosed by plasma basal cortisol values below 40 μg/L in neonates, and below 80 μg/L in older children, with no increase during hypoglycemia. Gonadotropin deficiency was diagnosed as the absence of clinical signs of puberty, undetectable plasma estradiol and testosterone concentrations, and no increase in gonadotropin level after a gonadotropin-releasing hormone test. The seasonal period evaluation was defined by the month of blood sampling, and we divided the patients into two groups: the sunny season, which was from June to September, and the cold season, which was from October to May.

Serum 25OHD and 1,25(OH)_2_D concentrations were measured using immunochemiluminescent assays (Liaison XL, DiaSorin, Saluggia, Italy). The patients were stratified according to their serum 25OHD concentration: ≤ 12 ng/mL, 13–19 ng/mL, 20–30 ng/mL, and > 30 ng/mL. We considered that a concentration between 30 and 60 ng/mL to be an appropriate level [9, 24, 25].

### Statistical analysis

Qualitative results are presented as percentages. Fisher’s exact test was performed to detect differences between qualitative variables. Quantitative results are presented as the mean ± SD. Between-group differences were assessed with the non-parametric Mann-Whitney test in cases with two groups and with the Kruskal-Wallis test in cases with 3 or more groups. Correlations were assessed with the Spearman test. A *p* value < 0.05 was considered significant. One boy with an abnormally high 25OHD concentration was excluded from the analysis because of probable vitamin D treatment before blood collection.

## Results

The mean serum 25OHD concentration was 33.2 ± 18.0 ng/mL. Two (4%), nine (18%), 15 (30%) and 24 (48%) patients had a 25OHD level ≤ 12 ng/mL, between 13 and 19 ng/mL, between 20 and 30 ng/mL, and above 30 ng/mL, respectively (Table 2). Among these, a concentration > 60 ng/mL was found for four patients. Sex and season were the two factors that significantly influenced 25OHD concentrations, with higher levels in boys than in girls and lower levels in the “cold” than in the “sunny” season (Tables 2 and 3). Conversely, no significant difference in 25OHD level was observed according to the isolated or multiple HP character deficiency, age, bone age, height, BMI, growth rate, GH peak level or IGF1 level.

**Table 2:** Characteristics according to the serum 25OHD level subgroups

25OHD, ng/mL	25OHD ≤ 12		13 < 25OHD ≤ 19		20 < 25OHD ≤ 30		25OHD > 30		
	*n* = 2		*n* = 9		*n* = 15		*n* = 24		*p*
Boys	0	0%	3	33%	7	47%	18	75%	0.04
Girls	2	100%	6	67%	8	53%	6	25%	
Isolated GH deficiency	1	50%	6	67%	4	27%	11	46%	0.2
Multiple HP deficiency	1	50%	3	33%	11	73%	13	54%	
Sunny season	0	0%	0	0%	4	27%	11	46%	0.01
Cold season	2	100%	6	100%	11	73%	13	54%	
	Mean ± SD		Mean ± SD		Mean ± SD		Mean ± SD		
Age at diagnosis, years	13.5 ± 4.5		4.7 ± 5.1		6.5 ± 6.7		4.4 ± 2.8		0.1
Bone age, years	12 ± 5		3.6 ± 5.6		3.2 ± 3.5		2.5 ± 1.9		0.1
Height, SDS	−2.7 ± −2.7		−2.6 ± 0.6		−3 ± 1.2		−2.8 ± 1.5		0.9
BMI, SDS	1.3 ± 0.8		0.4 ± 1.6		0.3 ± 2.3		0 ± 1.6		0.6
Growth rate, SDS	− 2.6 ± 0		− 2.4 ± 0.6		− 2.7 ± 1.2		− 3 ± 1.2		NS
IGF1, SDS	−4.7 ± 0		−2.4 ± 1.8		−3.9 ± 0.8		−3.6 ± 1		NS

**Table 3:** Serum 25OHD and 1,25(OH)_2_D levels compared with the patient characteristics

	25OHD, ng/mL		1,25(OH)_2_D, ng/mL	
	Mean ± SD	*p*	Mean ± SD	*p*
All PSIS (n = 50)	33.2 ± 18.0		74.5 ± 40.7	
Sex				
Boys (*n* = 28)	37.6 ± 18	0.04	70 ± 27	0.4
Girls (*n* = 22)	27.6 ± 17.3		75 ± 40	
Deficiency				
Isolated GH (*n* = 22)	32.4 ± 15	0.4	64 ± 11.9	0.1
Multiple HP (*n* = 28)	33.8 ± 20		83 ± 52	
Season				
Sunny (n = 15)	42 ± 18	0.03	71.5 ± 32	0.3
Cold (*n* = 35)	31 ± 13		73 ± 41	
Age, years				
≤ 1.5 years (n = 8)	31.4 ± 18	0.4	92 ± 17	0.5
1.5 < age ≤ 6 years (*n* = 23)	35.9 ± 19		73 ± 19	
6 < age ≤ 10 years (*n* = 13)	33.3 ± 17		74 ± 17	
10 < age ≤ 18 years (*n* = 6)	24.7 ± 9		58 ± 9	
BMI, SDS				
< 0 (*n* = 21)	33.2 ± 13	0.9	72 ± 36	0.8
≥ 0 (*n* = 25)	33 ± 20		69 ± 16	

The mean serum 1,25(OH)_2_D concentration was 74.5 ± 40.7 ng/L (Table 3). No significant difference in 1,25(OH)_2_D level was observed according to sex, isolated or multiple HP character deficiency, season, age, bone age, height, BMI and growth rate. However, we did observe a trend toward higher levels in those with multiple HP deficiency. No significant correlations were observed between serum 1,25 (OH)_2_D concentration and age, bone age, height, BMI, growth rate, IGF1 level or 25 OHD level. Nonetheless, significant positive correlations were observed between the GH peak level and serum 1,25 (OH)_2_D concentration (Rho = 0.35; *p* = 0.015) and the 1,25(OH)_2_D/25OHD ratio (Rho = 0.29; *p* < 0.05).

## Discussion

In the present study, we evaluated vitamin D status in a large series of children with GHD due to PSIS, as observed by a single endocrinologist. We performed this study because both GHD [26] and vitamin D deficiency [27] are associated with bone fragility, impaired growth, muscle weakness, cardiac outcomes and metabolic syndrome.

In our population, the mean 25OHD concentration was 33.2 ± 18.0 ng/mL, which appears to be a satisfactory level. As we did not include a control group, we compared this level with published data for healthy French children/adolescents. Among 212 girls aged 11–16.9 years with Tanner stages of 4 and 5 and living in Normandy (northwest of France), which is at the same latitude as Paris, mean 25OHD concentrations were 20.0 ± 6.1 ng/mL at the end of summer and 15.9 ± 7.4 ng/mL between January and May [28]. Alarmingly, 41% of these otherwise healthy girls had a 25OHD < 12 ng/mL during winter, revealing a severe vitamin D deficiency. The European HELENA study evaluated the vitamin D status of 1006 adolescents with a mean age of 14.9 years living in 10 European cities [29]. The participating French city was Lille, which is located in the extreme north of France, and the mean 25OHD level was 20.4 ± 8.8 ng/mL in girls and 24.1 ± 10.8 ng/mL in boys. In a recent multicenter study of vitamin D status in 326 healthy French children aged 6–10 years, it was found that 3.1% of the participants had a 25OHD concentration below 10 ng/mL and that 34.4% had a 25OHD concentration between 10 and 19.9 ng/mL [11]. Although the mean 25OHD values were not indicated in this article, it can be concluded that the vitamin D status of these children was quite similar to what we found in our children with PSIS. In our present study, female sex and winter season (from October to May) were significantly associated with lower serum 25OHD concentrations, which is consistent with other previous published studies in adult and pediatric healthy populations [9, 30].

We then compared the 25OHD concentration measured in the present study in our patients with PSIS to the values reported in recent studies on GHD patients from various countries prior to GH treatment. Saggese et al. evaluated 26 children with GHD [20] and found normal 25OHD concentrations but low 1,25 (OH)_2_D levels before GH treatment and a significant increase in 1,25 (OH)_2_D levels after 12 months of GH treatment. Ciresi et al. evaluated 80 Sicilian children with GHD [31]. These authors reported significantly higher values in the sunny season (31.1 ± 11.1 ng/mL in June–September) than in the cold season (17.3 ± 5.3 ng/mL in November–February), with 35% having vitamin D insufficiency and 40% vitamin D deficiency. Witkowska-Sedek et al. evaluated 84 children and adolescents with GHD and found low 25OHD concentrations (22.3 ± 6.9 ng/mL) [32], and Savanelli et al. evaluated 41 adult GHD patients and found mean 25OHD concentration of 21.3 ± 12.3 ng/mL, with vitamin D deficiency present in 51% of the patients compared to 14.6% of the controls [33]. In addition, Ameri et al. evaluated 69 adult GHD patients, most with postsurgical hypopituitarism [34], and found that only 6 patients (8.7%) had a serum 25OHD concentration above 30 ng/mL. These authors reported a positive association between vitamin D status and IGF1, and they also found a trend toward higher GH doses in patients with low vitamin D status (25OHD < 15 ng/mL), suggesting that a better vitamin D status may facilitate achieving normal IGF1 values in GHD patients. In a literature review, these authors proposed that assessment of vitamin D status might be an appropriate method of determining the dose of recombinant hGH for treatment of adult patients with GHD [34].

One possible explanation for the higher vitamin D status in our study may be related to the young age of the subjects (mean 5.4 years). Indeed, in France, the French Society of Paediatrics recommends systematic vitamin D supplementation of children up to five years of age. Unfortunately, no guidelines for older children have been implemented; an exception is adolescents, in whom winter supplementation is recommended but rarely performed [35]. Surprisingly, our patients of more than 5 years old, particularly those aged 6–9 years, had a mean 25OHD level similar to that of our younger patients. This may suggest that vitamin D supplementation had been offered to some of our older patients before evaluation in our unit. In support of this is the fact that although BMI is known to influence spontaneous vitamin D status with lower 25OHD concentrations in overweight subjects, we found no difference in 25OHD levels between patients with a BMI < 0 SDS and those with a higher BMI. If the hypothesis that some of our patients received vitamin D supplementation before their assessment is correct, the finding that they have a normal vitamin D status is likely independent of their GHD. However as no information on vitamin D supplementation before our evaluation was available in the medical charts of our patients, we were unable to confirm that their rather good vitamin D status was due to vitamin D supplementation.

As GH appears to increase the level of 1,25 (OH)_2_D [19, 20, 36, 37], albeit likely indirectly, through the effects of IGF1, it may be hypothesized that patients with GHD due to PSIS have a low 1,25 (OH) _2_D serum concentration. This was not the case in our patients who had normal levels. We, however, have no explanation for the higher 1,25(OH)2D level in those with multiple HP deficiencies. Regardless, we did find a significant positive correlation between the GH peak level obtained during a pharmacologic stimulation test and the 1,25 (OH) _2_D concentration as well as with the 1,25 (OH) _2_D/25OHD ratio. These findings suggest that even in these patients who had severely impaired GH secretion and low IGF1 levels, an interplay between the GH/IGF1 axis and the vitamin D system still exists.

Our study has limitations: it was retrospective and did not include a control group. However, the strength of our study pertains to the large series of GHD patients with a homogenous diagnosis (i.e., PSIS) and the same protocol of exploration for years, as they were attended by the same endocrinologist. This design allowed avoiding the considerable selection biases that affect several studies on GH deficiency. The patients who were excluded because of unavailable plasma samples might have introduced a bias, but their similarities to the included patients limit this. Another limitation is the lack of information on vitamin D supplementation before the patients were evaluated.

## Conclusions

We found quite an acceptable mean vitamin D status in our series of children with GHD due to PSIS. There are currently no available data from a double-blind, placebo-controlled randomized trial of vitamin D supplementation in GH-treated GHD patients showing that vitamin D optimizes bone health, growth and possibly other outcomes. Despite this lack of high-level evidence, we believe that it is mandatory to maintain optimal vitamin D supplementation in GHD patients.

## Abbreviations

1,25(OH)_2_D: 1,25-dihydroxyvitamin D
25OHD: 25-hydroxyvitamin D
BMI: Body mass index
GH: Growth hormone
GHD: Growth hormone deficiency
HP: Hypothalamic-pituitary
IGF1: Insulin-like growth factor 1
PSIS: Pituitary stalk interruption syndrome
SDS: Standard deviation score
